# Establishment of a Diet-Induced Systemic Metabolic SyndromeModel Reveals Early Renal Alterations and the Nephroprotective Potential of Three Jabuticaba (*Myrciaria cauliflora*) Formulations

**DOI:** 10.1007/s10735-026-10989-8

**Published:** 2026-10-15

**Authors:** Adriano Simões Barbosa Castro, Luiz Otávio Guimarães Ervilha, Francielle de Fátima Viana Santana, Renan do Carmo Lopes, Kelly Aparecida Dias, Lívya Alves Oliveira, Stephanie Michelin Santana Pereira, Kemilli Pio Gregório, Pedro Henrique Costa Neves, Mariella Bontempo Duca de Freitas, Renê Chagas da Silva, Ceres Mattos Della Lucia, Marli do Carmo Cupertino

**Affiliations:** 1 Structural Biology Laboratory, Department of General Biology, Universidade Federal de Viçosa, Viçosa, Minas Gerais State Brazil; 2 INSERM, C2VN (Cardiovascular and Nutrition Research Center), Aix-Marseille University, Marseille, France; 3 Laboratory of Vitamin Analysis, Department of Nutrition and Health, Universidade Federal de Viçosa, Viçosa, Minas Gerais State Brazil; 4 Chiropteran Ecophysiology Laboratory, Department of Animal Biology, Universidade Federal de Viçosa, Viçosa, Minas Gerais State Brazil; 5 Scanning Electron Microscopy Laboratory, Department of Physics, Universidade Federal de Viçosa, Viçosa, Minas Gerais State Brazil

**Keywords:** Metabolic syndrome, Kidney, *Myrciaria cauliflora*, Jabuticaba, Microencapsulation, Renal injury, Functional food

## Abstract

Metabolic syndrome is a progressive disorder associated with metabolic dysregulation, insulin resistance, chronic inflammation, and increased risk of chronic kidney disease. The kidney is an early target of metabolic imbalance, developing structural alterations before the establishment of advanced renal dysfunction. Jabuticaba (*Myrciaria cauliflora*), a Brazilian berry rich in phenolic compounds, has emerged as a promising functional food, and technological strategies such as microencapsulation may improve the stability and potential biological effects of its bioactive compounds. This study evaluated the effects of three jabuticaba formulations—freeze-dried whole fruit, freeze-dried peel, and microencapsulated peel extract—on renal morphology, relative elemental composition, and oxidative status in mice subjected to a high-fat, high-fructose (HFHF) diet. Fifty male C57BL/6 mice were allocated into five groups (*n* = 10): Control, HFHF, HFWF (HFHF + whole fruit), HFJP (HFHF + peel), and HFME (HFHF + microencapsulated peel extract). After six weeks of metabolic challenge, jabuticaba was introduced while maintaining the HFHF diet for an additional six weeks. At the end of the 12-week experimental period, kidneys were collected for morphometric, histopathological, elemental, and oxidative analyses. The HFHF diet induced early renal alterations characterized by increased vascular proportion, reduced tubular proportion, vascular congestion, tubular vacuolization, and inflammatory cell infiltration, indicating early renal structural changes associated with HFHF-induced metabolic dysfunction. Jabuticaba addition produced formulation-dependent effects on renal morphology, with the microencapsulated peel extract showing the most consistent histological preservation. The HFHF diet also altered the renal elemental profile, increasing the relative proportions of calcium and manganese and reducing sodium compared with Control animals. Among the Jabuticaba groups, HFWF shifted calcium and sodium proportions toward Control values, whereas HFJP and HFME maintained the HFHF-associated pattern for these elements. For manganese, HFJP and HFME showed intermediate values that did not differ significantly from either Control or HFHF animals. No significant differences were observed among groups for oxidative stress markers (malondialdehyde, protein carbonyls, nitric oxide, and FRAP) or antioxidant enzymes (catalase, superoxide dismutase, and glutathione S-transferase), indicating that the observed morphological and elemental disturbances were not accompanied by detectable changes in renal oxidative stress biomarkers or antioxidant defenses. Thus, the effects associated with jabuticaba should not be interpreted as primarily antioxidant-mediated. These findings indicate that *Myrciaria cauliflora* addition in HFHF diet was associated mainly with preservation of renal histological architecture and modulation of the relative elemental profile in a formulation-dependent manner, with the microencapsulated peel extract showing the most consistent histological effects. The results highlight the potential of jabuticaba peel valorization and microencapsulation technology as strategies for developing functional ingredients aimed at preserving renal tissue during early metabolic dysfunction, although further functional and molecular studies are required to confirm nephroprotective effects.

## Highlights

- HFHF feeding for 12 weeks successfully induced a systemic metabolic syndrome phenotype in mice, characterized by increased blood glucose, cholesterol, and triglycerides, validating the experimental model.
- Systemic metabolic syndrome induced early renal structural alterations, including vascular congestion, tubular vacuolization, inflammatory infiltration, increased vascular proportion, and reduced tubular proportion.
- Jabuticaba addition to the HFHF diet preserved renal architecture, attenuating histological changes and restoring tubular and vascular parameters, with the microencapsulated peel extract showing the most consistent protective effect.
- Metabolic syndrome altered renal mineral homeostasis, increasing calcium and manganese levels and reducing sodium concentration, while potassium, magnesium, zinc, copper, and iron remained unchanged.
- Jabuticaba formulations differentially modulated renal mineral balance, with peel addition in HFHF diet restoring calcium and sodium levels and whole fruit and microencapsulated extract normalizing manganese concentration.
- Renal injury begins before overt kidney dysfunction, and jabuticaba may delay the progression through structural and metabolic preservation rather than through a classical antioxidant mechanism.

## Introduction

Metabolic syndrome (MetS) has become a major global public health challenge, affecting nearly 30% of the adult population worldwide and continuing to increase in prevalence. Rather than a single disease, MetS comprises a cluster of interconnected metabolic abnormalities, including obesity, insulin resistance, dyslipidemia, hypertension, and impaired glucose metabolism (Fahed et al. 2022; World Health Organization 2025). Importantly, metabolic disturbances may initiate tissue remodeling and cellular injury before overt clinical manifestations become evident, emphasizing the importance of identifying early pathological events and preventive strategies (Ruiz-García et al. 2025; Obeidat et al. 2025).

Among the organs affected during MetS progression, the kidney is particularly susceptible because of its high metabolic activity, continuous exposure to circulating metabolites, and tightly regulated hemodynamic and endocrine functions (Romeo et al., 2025). Experimental and clinical evidence indicates that renal injury may begin early during metabolic dysfunction, preceding clinically detectable chronic kidney disease (CKD) (Francis et al. 2024; Stasi et al. 2022). Early renal alterations include vascular congestion, tubular epithelial injury, inflammatory cell infiltration, glomerular remodeling, and disturbances in mineral homeostasis, which may contribute to progressive nephron dysfunction (Mingyue & JIN 2021; Kovesdy 2025). High-fat, high-fructose (HFHF) diets reproduce several of these alterations in experimental models, even before severe obesity or advanced renal impairment develops (Yustisia et al. 2022; Bier et al. 2022; Kume et al. 2007). However, strategies capable of preventing or attenuating these early renal lesions remain insufficiently explored.

In this context, plant-derived bioactive compounds have gained attention as potential nutritional strategies for preventing metabolic-related tissue injury (Pelczyńska et al. 2023; Pérez-Torres et al. 2021). Among Brazilian native fruits, jabuticaba (*Myrciaria cauliflora*) is particularly rich in anthocyanins, ellagitannins, flavonoids, and other phenolic compounds, as well as dietary fiber, vitamins, and essential minerals (Nascimento et al. 2025; Bocker and Silva 2024). These bioactive constituents have been associated with antioxidant, anti-inflammatory, and metabolic benefits in experimental and clinical studies (Batista et al. 2014; De Araújo et al. 2025). Notably, a recent clinical study suggested the potential of jabuticaba peel supplementation as an adjunct nutritional strategy for patients with chronic kidney disease (Lima et al. 2025). Nevertheless, its direct effects on renal tissue during early metabolic dysfunction remain poorly understood, particularly whether different jabuticaba preparations provide distinct degrees of renal protection.

Most available studies have focused on jabuticaba peel because of its particularly high phenolic content, whereas direct comparisons between whole-fruit and peel preparations remain scarce, and their relative ability to preserve renal structure remains unclear. Moreover, the limited stability and gastrointestinal bioavailability of phenolic compounds may restrict their biological efficacy. Microencapsulation can protect anthocyanins and other polyphenols from degradation and promote controlled release, potentially improving their bioavailability and biological effects (Loubet Filho et al. 2022; Oliveira et al. 2024; Pauletto et al. 2025). Thus, directly comparing whole fruit, phenolic-rich peel, and microencapsulated peel extract provides an opportunity to determine whether food matrix composition and technological processing influence their renal effects. The use of jabuticaba peel also contributes to the valorization of agro-industrial by-products, providing a sustainable source of bioactive compounds for functional foods and animal nutrition (Soares et al. 2018; Nascimento et al. 2025).

Therefore, the present study investigated whether dietary addition with freeze-dried whole jabuticaba, freeze-dried jabuticaba peel, or microencapsulated jabuticaba peel extract on HFHF diet could attenuate early kidney injury induced by a high-fat, high-fructose diet. Renal protection was evaluated through histomorphological, oxidative, and mineral analyses of the renal cortex. By directly comparing these three preparations under the same experimental conditions, this study evaluates whether the whole-food matrix, phenolic-rich peel, and microencapsulation result in distinct patterns of renal preservation during early metabolic dysfunction.

## Materials and methods

### Plant material and preparation of jabuticaba fractions

Fruits of *Myrciaria cauliflora* were obtained during the harvest season from rural producers located in Jequeri, Minas Gerais, Brazil. After collection, fruits were washed, manually selected, and separated into two fractions: whole fruit and peel. Both fractions were freeze-dried and subsequently ground using a knife mill until a homogeneous powder was obtained. The resulting powders were stored under controlled conditions until incorporation into the experimental diets. Data regarding the collection, processing, and composition of the jabuticaba fruit, as well as the microencapsulation process, were described in detail by Dias et al. (2026).

### Preparation of microencapsulated jabuticaba peel extract

The microencapsulated extract was produced from previously freeze-dried jabuticaba peel flour. A hydroethanolic extraction was performed, and the extract was subsequently filtered, concentrated under reduced pressure using a rotary evaporator coupled to a vacuum system, and dried by spray drying (Dias et al. 2026).

Maltodextrin, inulin, whey protein concentrate (WPC), and lactose were used as encapsulating agents at a concentration of 30% and previously diluted in distilled water. The microencapsulated extracts were stored in hermetically sealed containers protected from light at − 20 °C until dietary formulation.

The amount of microencapsulated extract incorporated into the diet was calculated to provide 200 mg of polyphenols per kg of body weight, according to previous studies (Pauletto et al. 2025). Microencapsulation was selected as a technological strategy to improve the stability of jabuticaba phenolic compounds and potentially enhance their bioavailability (Dias et al. 2026).

### Animals and experimental design

Fifty male C57BL/6 mice, eight weeks old, were obtained from the Central Animal Facility of the Center for Biological and Health Sciences at the Federal University of Viçosa (UFV), Brazil. Animals were housed collectively (five animals per cage) in polypropylene cages with metal grid covers (330 × 200 × 130 mm) under controlled environmental conditions (21 ± 3 °C; 12 h light/dark cycle). Animals had free access to water and diets throughout the experimental period and underwent a one-week acclimatization period before experimentation.

All experimental procedures were approved by the Ethics Committee on Animal Use of the Federal University of Viçosa (protocol 10/2023) and followed the guidelines established by the Brazilian National Council for Control of Animal Experimentation (CONCEA).

The experimental protocol consisted of two phases. During phase I, metabolic alterations were induced. Animals were randomly divided into two groups: Control (*n* = 10), receiving the AIN-93 M diet according to the recommendations of the American Institute of Nutrition (Reeves et al. 1993), and HFHF (*n* = 40), receiving a high-fat, high-fructose diet. Metabolic alterations were induced for six weeks according to a previously described experimental protocol (Marineli et al. 2015). After this induction period, animals were allocated to the Jabuticaba groups while maintaining the HFHF diet for an additional six weeks.

During phase II, Control animals continued receiving the AIN-93 M diet, whereas HFHF animals were redistributed according to body weight into four experimental groups, with 10 animals per group (*n* = 10/group; total *n* = 50):

- HFHF: High-fat, high-fructose diet (*n* = 10).
- HFWF: HFHF diet with freeze-dried whole jabuticaba (*n* = 10).
- HFJP: HFHF diet with freeze-dried jabuticaba peel (*n* = 10).
- HFME: HFHF diet with microencapsulated jabuticaba peel extract (*n* = 10).

The Jabuticaba’s experimental period lasted six weeks, resulting in a total experimental period of 12 weeks. Jabuticaba addition in HFHF diet was initiated after the initial six-week HFHF induction period to investigate the effects of jabuticaba preparations after the onset of diet-induced metabolic alterations rather than their effects when administered concomitantly with HFHF exposure from the beginning of the experiment.

### Dietary formulation and jabuticaba preparation

The HFHF diet contained 60% lipids and 20% fructose. After nutritional characterization of the fruit, considering carbohydrate, protein, lipid, and fiber contents, the jabuticaba formulations were incorporated into the experimental diets at amounts calculated to provide 200 mg of polyphenols per 100 g of diet. Whole jabuticaba powder was incorporated at 6.4 g/100 g of diet (64 g/kg of HFHF diet), lyophilized and milled jabuticaba peel at 3.4 g/100 g of diet (34 g/kg of HFHF diet), and microencapsulated jabuticaba peel extract at 20.3 g/100 g of diet (203 g/kg of HFHF diet). Ingredient proportions were adjusted to maintain equivalent macronutrient composition and energy density among the experimental groups (Abot et al. 2022; Fialho et al. 2025).

All diet ingredients were weighed using a semi-analytical balance (Gehaka BG2000, Brazil), manually mixed, sieved, homogenized in an industrial mixer (Leme^®^, Brazil), manually pelletized, and stored at − 18 ± 1 °C until use.

### Sample collection and renal tissue preparation

At the end of the 12-week experimental period, animals were subjected to a 12 h fasting period and euthanized under deep inhalational anesthesia with isoflurane (3%; Isoforine^®^, Cristália, Brazil). The abdominal cavity was opened, and kidneys were collected, weighed, and washed with 0.9% saline solution.

The right kidneys were stored at − 80 °C for oxidative stress analyses. The left kidneys were fixed by immersion in 3.7% formaldehyde for 48 h and subsequently stored in 70% ethanol until histological processing.

The renal somatic index was calculated by normalizing the total kidney weight to final body weight.

### Histological processing and morphometric analysis

Renal cortical fragments were processed using routine histological procedures, embedded in paraffin, and sectioned using a rotary microtome (RM 2255, Leica Biosystems, Nussloch, Germany). Sections of 5 μm thickness were obtained using a semi-serial sampling approach, in which one section from every ten consecutive sections was analyzed to avoid repeated evaluation of the same tissue area.

Sections were stained with hematoxylin and eosin (H&E) for evaluation of renal architecture and morphometric parameters, Picrosirius Red for collagen fiber visualization, and periodic acid–Schiff (PAS) staining for evaluation of mesangial matrix alterations and glomerular structural changes.

Digital images of renal cortex areas were captured at 20 × and 40 × magnification using a bright-field microscope (Olympus BX53, Tokyo, Japan) equipped with a digital camera (Olympus DP73, Tokyo, Japan). Image analysis was performed using Image-Pro Plus^®^ 4.5 software (Media Cybernetics, Silver Spring, USA).

Morphometric and stereological analyses were performed with the observer blinded to the experimental groups using a 266-point grid system. Ten randomly selected microscopic fields per animal were evaluated. The volumetric proportion of renal structures was determined according to the formula:

$$\% \;{\mathrm{component}}={\mathrm{PP/PT}} \times 100$$

where PP represents the number of points coinciding with the evaluated structure and PT represents the total number of test points (Freitas et al. 2020).

### Histopathological evaluation

Histopathological examination was performed to identify early kidney injury alterations associated with metabolic syndrome. The evaluated parameters included vascular congestion, tubular epithelial vacuolization, inflammatory infiltration, and structural alterations in renal compartments.

Ten randomly selected cortical fields per animal were analyzed under light microscopy. The histopathological evaluation was performed blinded to experimental group allocation.

The area of renal corpuscles, glomeruli, and Bowman’s space was determined in 20 randomly selected glomeruli per animal using Image-Pro Plus^®^ software. Bowman’s space area was calculated by subtracting glomerular area from renal corpuscle area.

### Renal mineral analysis

Renal cortex fragments (~ 5 mm) were dried at 60 °C for 96 h, carbon-coated (Quorum Q150T), and analyzed by scanning electron microscopy coupled with energy-dispersive X-ray spectroscopy (SEM-EDS; JEOL JSM-6010LA).

Analyses were performed at 150 × magnification, using an accelerating voltage of 20 kV and a working distance of 19 mm. The concentrations of copper (Cu), iron (Fe), manganese (Mn), zinc (Zn), calcium (Ca), magnesium (Mg), sodium (Na), and potassium (K) were determined and expressed as the percentage of elemental composition in renal tissue.

SEM-EDS provides the relative elemental composition of the analyzed tissue area rather than absolute mineral concentrations. Therefore, the results were expressed as the relative percentage of each detected element and should be interpreted as semi-quantitative estimates of renal elemental composition rather than absolute tissue mineral concentrations.

### Oxidative stress biomarkers

Renal samples (100 mg), previously stored at − 80 °C, were homogenized in 0.2 M phosphate buffer containing 1 mM EDTA (pH 7.4). Homogenates were centrifuged at 15,000 × g for 10 min at 4 °C, and the supernatants were used for biochemical analyses.

Oxidative stress markers included malondialdehyde (MDA), protein carbonyl content (PC), nitric oxide (NO), and total antioxidant capacity (FRAP). Protein carbonyl levels were determined according to Levine et al. (1990), and lipid peroxidation was evaluated by the thiobarbituric acid reactive substances (TBARS) assay according to Buege and AUST (1978). Total antioxidant capacity was determined by the ferric reducing antioxidant power (FRAP) assay, and nitric oxide levels were quantified by the Griess reaction.

Total protein content was determined using the Bradford assay (Bradford 1976), and biochemical results were normalized according to protein concentration.

### Antioxidant enzyme activities

Antioxidant enzyme activities were determined as previously described by Santana et al. (2023). Superoxide dismutase (SOD), catalase (CAT), and glutathione S-transferase (GST) activities were assessed according to Dieterich et al. (2000), Goth (1991) and Korolyuk et al. (1988), with modifications by Hadwan and Abed (2016), and Habig et al. (1974), respectively.

Enzyme activities were normalized according to total protein concentration and expressed as enzymatic units per milligram of protein.

### Statistical analysis

Data distribution was evaluated using the Shapiro–Wilk test. Statistical comparisons were performed using one-way analysis of variance (ANOVA), followed by Tukey’s post hoc test.

Differences were considered statistically significant when *p* < 0.05. Statistical analyses and graphs were generated using GraphPad Prism version 8.0 (GraphPad Software Inc., San Diego, CA, USA). Data are presented as mean ± standard deviation.

## Results

Metabolic syndrome was confirmed at the sixth week by an increase in caudal blood glucose levels, and at the end of the 12th week by an increase in blood glucose and serum triglycerides.

### Renal biometric parameters

Renal biometric parameters are presented in Table 1. No significant differences were observed among experimental groups regarding absolute kidney weight or renal somatic index (*p* > 0.05). These findings indicate that the induction of metabolic syndrome by a high-fat high-fructose diet did not result in changes in overall renal mass during the experimental period.

**Table 1:** Renal weight and renal somatic index of mice with metabolic syndrome subjected to a high-calorie diet and different jabuticaba formulations

Parameters	Control	HFHF	HFWF	HFJP	HFME
Kidney weight (g)	0.26 ± 0.02	0.28 ± 0.02	0.27 ± 0.01	0.26 ± 0.03	0.28 ± 0.02
Renal somatic index	1.10 ± 0.16	1.09 ± 0.13	1.06 ± 0.73	1.04 ± 0.26	1.14 ± 0.24

Data are expressed as mean ± standard deviation. Control: AIN-93 M standard rodent diet; HFHF: high-fat high-fructose diet; HFWF: HFHF diet with freeze-dried whole jabuticaba; HFJP: HFHF diet with freeze-dried jabuticaba peel; HFME: HFHF diet supplemented with microencapsulated jabuticaba peel extract.

### Renal morphometric and histopathological analysis

**Fig. 1:**
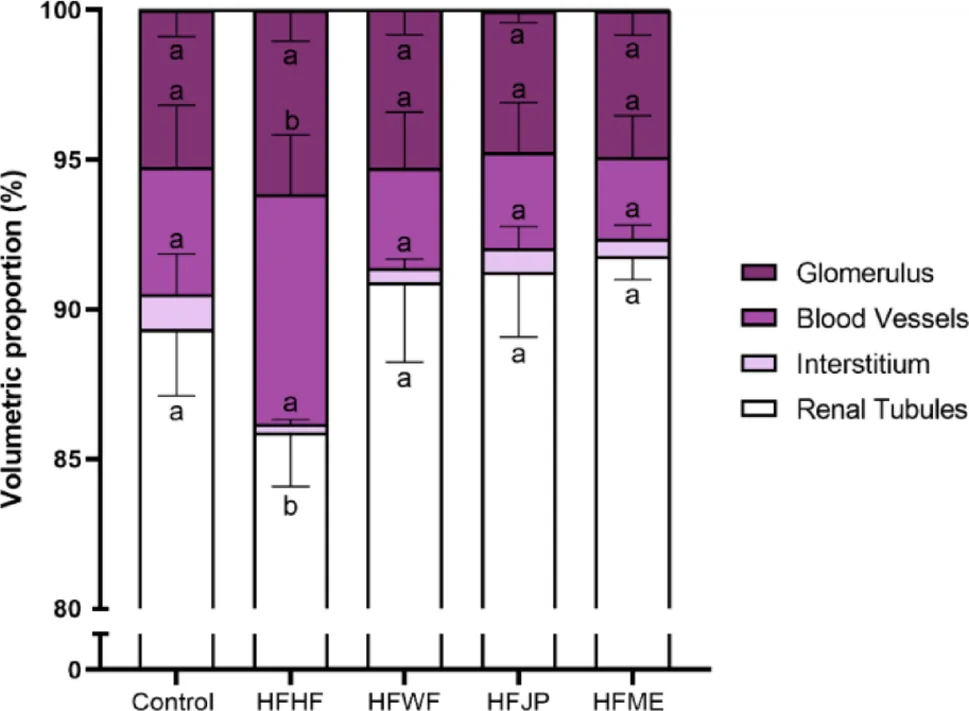
Volumetric proportion (%) of renal cortical compartments (glomeruli, blood vessels, interstitium, and renal tubules) in experimental groups (Control, HFHF, HFWF, HFJP, and HFME). Control, AIN-93 M standard rodent diet; HFHF, high-fat high-fructose diet; HFWF, HFHF diet with freeze-dried whole jabuticaba; HFJP, HFHF diet with freeze-dried jabuticaba peel; HFME, HFHF diet with microencapsulated jabuticaba peel extract. Different superscript letters indicate significant differences among groups (*p* < 0.05) according to one-way ANOVA followed by Tukey’s test (*n* = 10 animals/group)

Morphometric analysis revealed that HFHF feeding induced early structural alterations in the renal cortex, characterized by an approximately 3,48% reduction in tubular volumetric proportion and an approximately 3,69% increase in blood vessel proportion compared with the Control group (*p* < 0.05; Fig. 1). Notably, all jabuticaba-Jabuticaba groupss (HFWF, HFJP, and HFME) showed tubular and vascular proportions comparable to Control values and significantly different from those of the HFHF group (*p* < 0.05). No significant differences were observed among groups in glomerular or interstitial proportions (*p* > 0.05).

### Renal morphometric and histopathological analysis (continued)

**Fig. 2:**
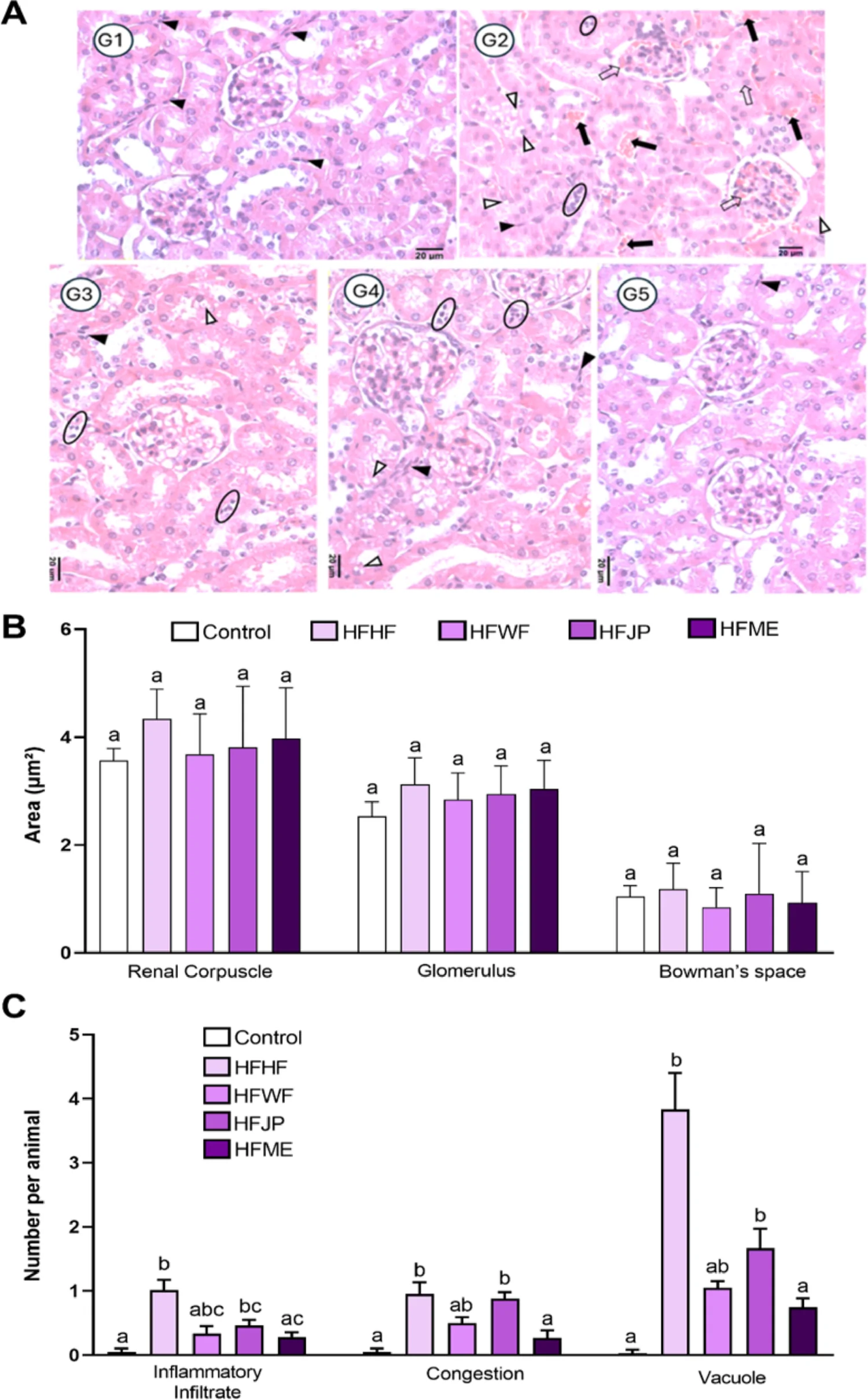
Representative photomicrographs of renal cortex sections stained with hematoxylin and eosin and renal morphometric parameters of experimental groups G1, G2, G3, G4, and G5 (Control, HFHF, HFWF, HFJP, and HFME). **A** Representative renal cortex images from Control, HFHF, HFWF, HFJP, and HFME groups. Scale bar = 20 μm. Arrows indicate vascular congestion (thick black arrows), mononuclear inflammatory infiltration (ellipses), tubular epithelial vacuolization (white arrowheads), interstitial region (black arrowhead), and erythrocytes within glomerular capillaries (thick white arrows). **B** Morphometric analysis of renal corpuscle area, glomerular area, and Bowman’s space area (µm^2^). **C** Quantification of histopathological alterations, including inflammatory infiltration, vascular congestion, and tubular vacuolization per histological field

Microscopic evaluation of hematoxylin and eosin (H&E)-stained sections revealed preserved renal cortical architecture in Control animals, characterized by well-defined glomeruli, intact Bowman’s capsules, preserved urinary spaces, and normal organization of tubular, interstitial, and vascular compartments. No relevant structural abnormalities or tubular epithelial vacuolization were observed, confirming tissue integrity (Fig. 2A).

In contrast, kidneys from HFHF animals exhibited early histopathological alterations associated with metabolic stress. The renal cortex showed vascular dilation and congestion in both interstitial vessels and glomerular capillaries. Additionally, proximal tubular epithelial cells presented cytoplasmic vacuolization, suggestive of intracellular lipid accumulation. The interstitial compartment exhibited mild mononuclear inflammatory infiltration associated with vascular congestion (Fig. 2A).

Histopathological semi-quantitative analysis showed that HFHF animals exhibited a higher frequency of inflammatory infiltration, vascular congestion, and tubular vacuolization than Control animals (Fig. 2C). In HFHF animals, inflammatory infiltration and vascular congestion were observed at an average frequency of approximately 1 occurrence per animal, whereas tubular vacuolization reached approximately 4 occurrences per animal, compared with nearly absent alterations in Control animals. Among the Jabuticaba groupss, HFME showed the most consistent histopathological response, with all three parameters significantly lower than in HFHF animals and comparable to Control values. HFWF showed an intermediate response, with no significant differences from either Control or HFHF animals, whereas HFJP remained statistically comparable to HFHF for all three parameters.

No significant differences were observed among experimental groups regarding renal corpuscle area, glomerular area, or Bowman’s space area (Fig. 2B), indicating that the metabolic challenge affected mainly tubular and vascular compartments rather than glomerular size.

Data are expressed as mean ± standard deviation. Different letters indicate significant differences among groups (*p* < 0.05), determined by one-way ANOVA followed by Tukey’s post hoc test (*n* = 10 animals/group).

### PAS staining analysis

**Fig. 3:**
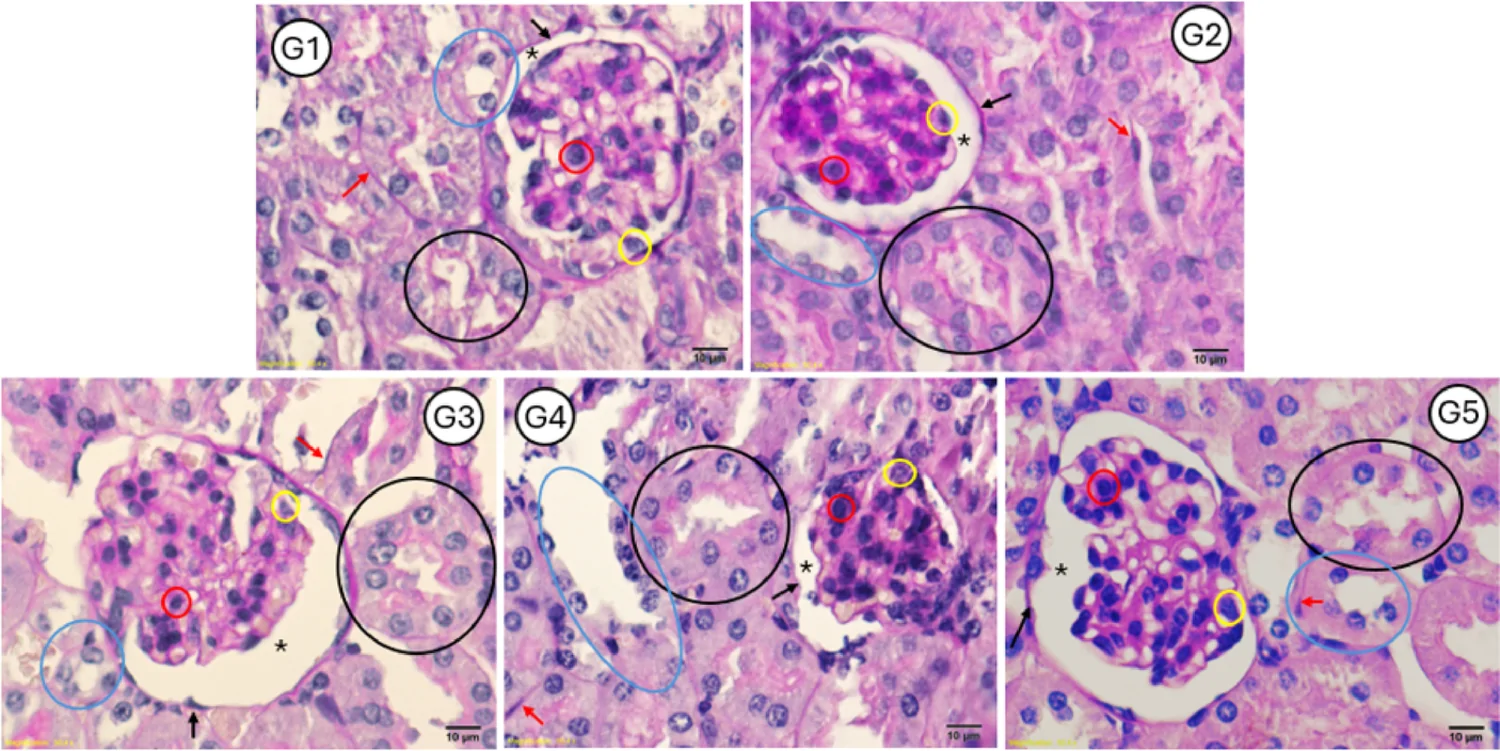
Representative photomicrographs of renal cortex sections stained with periodic acid–Schiff (PAS) from experimental groups (Control, HFHF, HFWF, HFJP, and HFME). PAS staining showing renal structures, including glomerular capsule (black arrow), interstitium (red arrow), urinary space (asterisk), podocytes (yellow circle), mesangial cells (red circle), proximal convoluted tubule (black circle), and distal convoluted tubule (blue circle). Experimental groups: G1 Control (AIN-93 M standard diet); G2 HFHF (high-fat high-fructose diet); G3 HFWF (HFHF + freeze-dried whole jabuticaba); G4 HFJP (HFHF + freeze-dried jabuticaba peel); G5 HFME (HFHF + microencapsulated jabuticaba peel extract). Scale bar = 10 μm. *n* = 10 animals/group

PAS staining revealed greater PAS-positive mesangial areas in HFHF animals compared with Control animals, consistent with increased mesangial matrix deposition (Fig. 3). Similar PAS-positive changes were observed in the HFJP group. In contrast, HFWF and HFME animals showed PAS staining patterns visually comparable to those of Control animals.

The increased PAS-positive material within the glomerular mesangial regions is consistent with expansion/deposition of carbohydrate-rich extracellular matrix components. Because this assessment was based on histological PAS staining rather than specific molecular markers of individual extracellular matrix components, the finding is described as increased PAS-positive mesangial matrix rather than as deposition of a specific matrix protein.

### Picrosirius red staining analysis

**Fig. 4:**
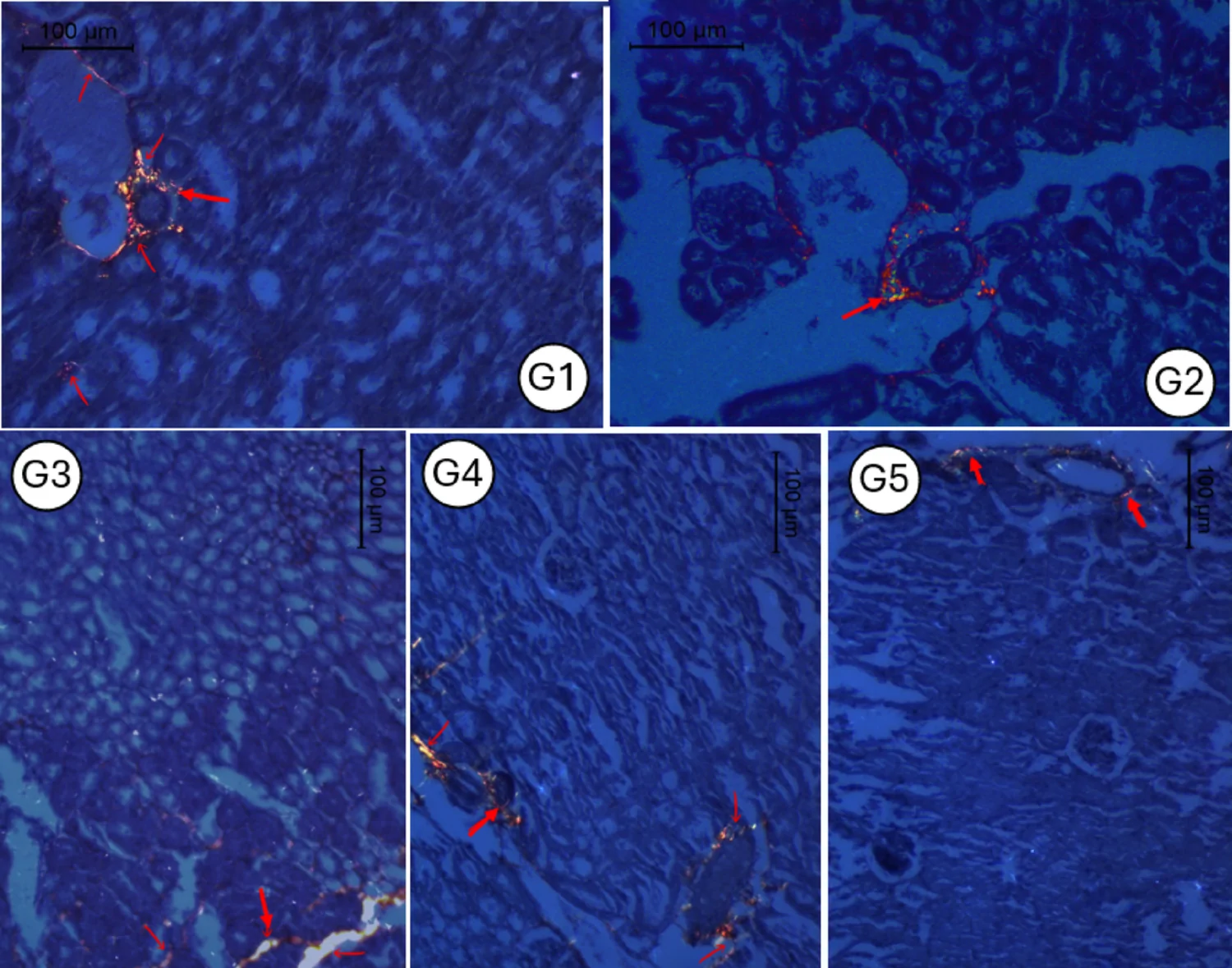
Representative photomicrographs of renal cortex sections stained with Picrosirius Red for collagen evaluation. Collagen fibers are indicated by red staining under bright-field microscopy or birefringence under polarized light. Experimental groups: G1 Control (AIN-93 M standard diet); G2 HFHF (high-fat high-fructose diet); G3 HFWF (HFHF + freeze-dried whole jabuticaba); G4 HFJP (HFHF + freeze-dried jabuticaba peel); G5 HFME (HFHF + microencapsulated jabuticaba peel extract). Scale bar = 100 μm. *n* = 10 animals/group

No detectable increase in collagen deposition was observed in HFHF animals or in any jabuticaba-Jabuticaba groups compared with Control animals (Fig. 4). Thus, the structural alterations detected by H&E and PAS staining in HFHF animals were not accompanied by detectable collagen accumulation during the 12-week experimental period.

The absence of increased Picrosirius Red staining in HFHF animals indicates that the renal alterations observed at this experimental stage had not progressed to detectable collagen-rich fibrotic remodeling.

### Renal mineral profile

**Fig. 5:**
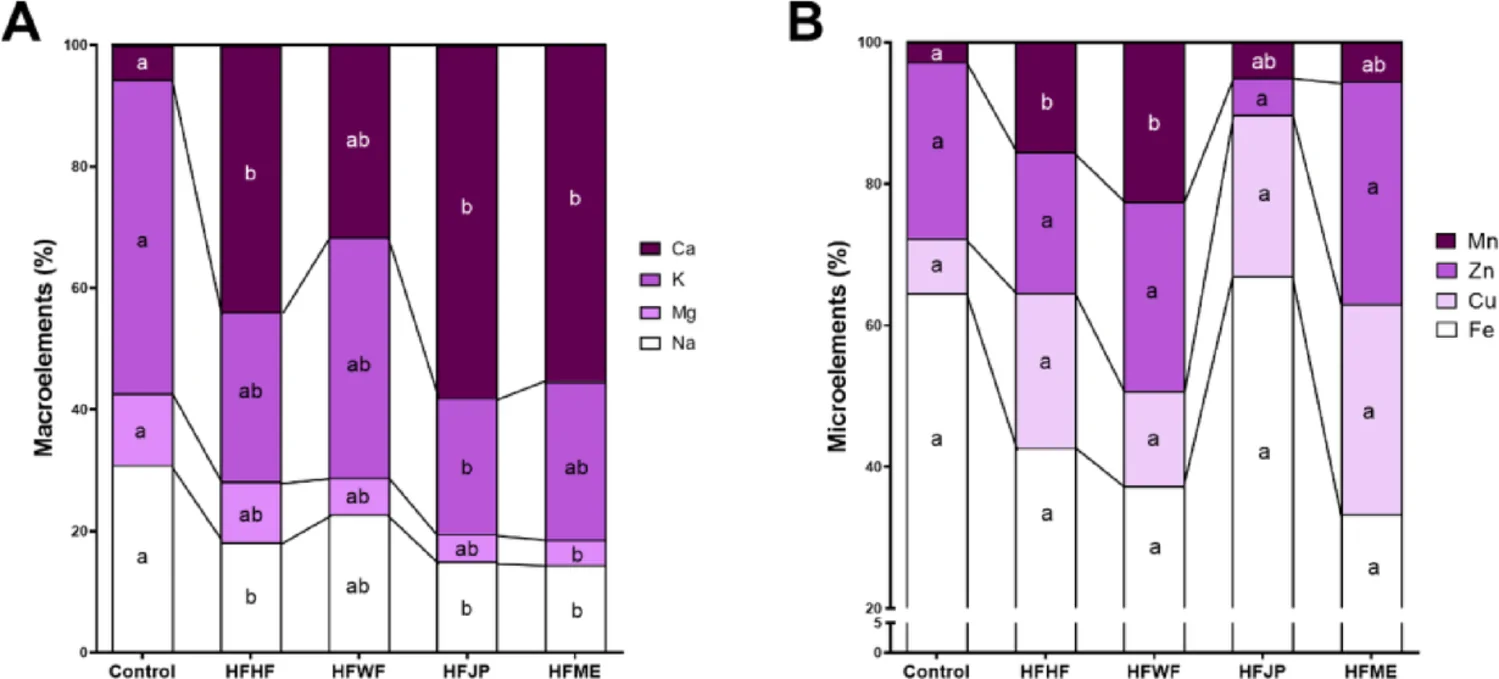
Renal macro- and micromineral profile of mice subjected to different dietary treatments. Percentage composition of macrominerals calcium (Ca), potassium (K), magnesium (Mg), and sodium (Na), and microminerals manganese (Mn), zinc (Zn), copper (Cu), and iron (Fe) in renal cortical tissue. Experimental groups: Control (AIN-93 M standard diet); HFHF (high-fat high-fructose diet); HFWF (HFHF + freeze-dried whole jabuticaba); HFJP (HFHF + freeze-dried jabuticaba peel); HFME (HFHF + microencapsulated jabuticaba peel extract). Different superscript letters indicate significant differences among groups (*p* < 0.05), according to one-way ANOVA followed by Tukey’s test (*n* = 10 animals/group).

SEM-EDS analysis revealed differences in the relative elemental composition of renal cortical tissue among experimental groups (Fig. 5). HFHF animals exhibited a significantly higher relative proportion of Ca and a lower relative proportion of Na compared with Control animals (*p* < 0.05). Among the Jabuticaba groupss, HFWF shifted the relative proportions of Ca and Na toward Control levels, resulting in values that were not significantly different from those of Control animals. In contrast, HFJP and HFME maintained the HFHF-associated pattern for both elements.

Regarding trace elements, HFHF animals exhibited a significantly higher relative proportion of Mn than Control animals (*p* < 0.05). HFWF showed a Mn proportion comparable to HFHF, whereas HFJP and HFME exhibited intermediate values that did not differ significantly from either Control or HFHF animals. No significant differences were detected among groups in the relative proportions of K, Mg, Zn, Cu, or Fe (*p* > 0.05).

Because SEM-EDS provides relative elemental composition rather than absolute tissue mineral concentrations, these findings represent semi-quantitative differences in the elemental profile of the analyzed renal tissue.

### Oxidative stress markers and antioxidant enzyme activities

**Fig. 6:**
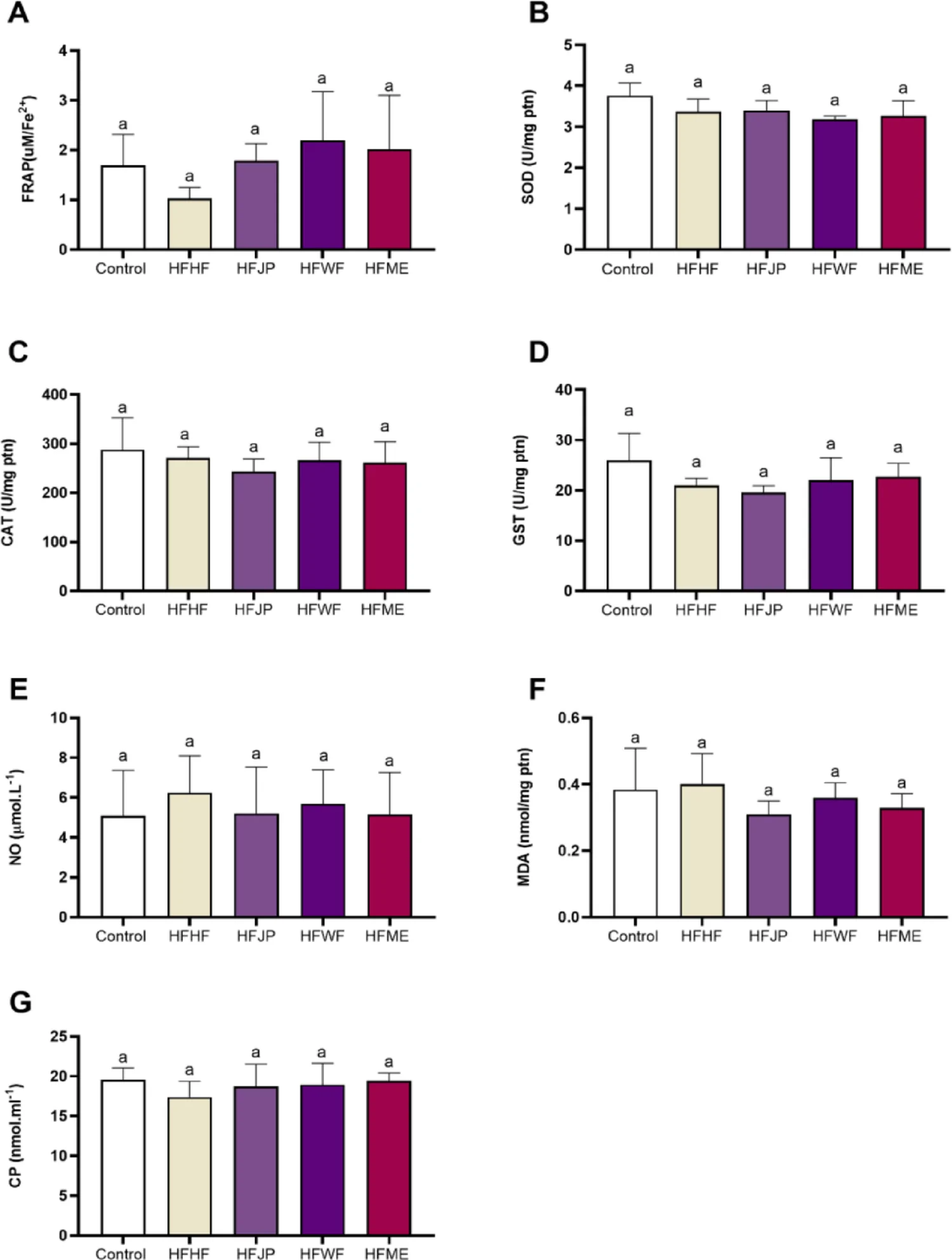
Effects of high-fat high-fructose diet and jabuticaba addition in HFHF diet on renal oxidative status and antioxidant enzyme activities. **A** Total antioxidant capacity (FRAP). **B** Superoxide dismutase activity (SOD). **C** Catalase activity (CAT). **D** Glutathione S-transferase activity (GST). **E** Nitric oxide levels (NO). **F** Malondialdehyde levels (MDA). **G** Protein carbonyl content (PC). Experimental groups: Control (AIN-93 M standard diet); HFHF (high-fat high-fructose diet); HFWF (HFHF + freeze-dried whole jabuticaba); HFJP (HFHF + freeze-dried jabuticaba peel); HFME (HFHF + microencapsulated jabuticaba peel extract). Data are expressed as mean ± standard deviation. Different superscript letters indicate significant differences among groups (*p* < 0.05), according to one-way ANOVA followed by Tukey’s post hoc test (*n* = 10 animals/group)

No significant differences were detected among experimental groups for any of the evaluated oxidative stress or antioxidant parameters (*p* > 0.05; Fig. 6). Specifically, FRAP, SOD, CAT, GST, NO, MDA, and protein carbonyl content remained statistically similar among Control, HFHF, HFWF, HFJP, and HFME groups.

Thus, despite the renal morphological and histopathological alterations observed in HFHF animals, these changes were not accompanied by detectable differences in the evaluated oxidative stress biomarkers or antioxidant enzyme activities during the experimental period.

## Discussion

Metabolic syndrome is a progressive condition in which early metabolic disturbances may affect target organs before the establishment of overt renal dysfunction or chronic kidney disease. The kidney is particularly susceptible to metabolic overload because of its high energy demand, complex microvascular regulation, and continuous exposure to circulating metabolic alterations. However, the initial renal responses triggered by a high-calorie diet, particularly during the subclinical phase of metabolic dysfunction, remain insufficiently characterized. In this context, the present study aimed to characterize the early renal alterations induced by a high-fat, high-fructose diet and to investigate the potential protective effects of three distinct forms of *Myrciaria cauliflora* addition in HFHF diet: whole fruit, representing the complete food matrix; peel, an agro-industrial by-product enriched in bioactive compounds; and a microencapsulated peel extract, representing a technological approach designed to improve the stability and potential bioavailability of jabuticaba-derived compounds. By directly comparing these formulations, this study provides insights into how food matrix composition and technological processing may influence renal responses during the early stages of metabolic dysfunction.

The present findings demonstrate that the HFHF challenge induced early renal structural alterations without detectable changes in the evaluated oxidative stress biomarkers or advanced fibrotic remodeling. HFHF-fed animals exhibited vascular congestion, mild inflammatory infiltration, tubular epithelial vacuolization, and changes in the relative distribution of renal cortical compartments. In contrast, kidney weight, renal somatic index, glomerular morphometry, and collagen deposition remained unchanged. Together, these findings are consistent with an early stage of renal involvement in which structural alterations are already detectable but have not yet progressed to extensive glomerular remodeling or collagen-rich fibrosis. However, whether these alterations are functionally reversible cannot be established from the present experimental design.

The vascular and tubular alterations observed in the present study may represent early renal responses to metabolic imbalance. Renal microcirculation depends on endothelial integrity and precise regulation of vascular tone, whereas proximal tubular cells have high metabolic demands due to their intense transport activity and mitochondrial dependence. Circulating free fatty acids can exceed the kidney’s lipid-metabolizing capacity, resulting in lipid accumulation particularly in tubular epithelial cells and contributing to mitochondrial dysfunction, inflammation, and cellular injury (Hughes et al. 2025). Indeed, microvascular dysfunction and metabolic stress in renal cell populations, have been described as early features of MetS-associated kidney injury, preceding more advanced structural damage (Marshall et al. 2026). Thus, the vascular congestion, mild inflammatory infiltration, and tubular vacuolization observed herein are consistent with early renal remodeling associated with HFHF-induced metabolic dysfunction. However, because endothelial markers, inflammatory cytokines, and molecular indicators of tubular injury were not evaluated, the mechanisms underlying these histological alterations cannot be directly established.

A particularly relevant finding was the dissociation between renal structural alterations and oxidative stress biomarkers. Despite the histological changes observed in HFHF animals, no significant differences were detected in MDA, protein carbonyl content, NO, FRAP, SOD, CAT, or GST among experimental groups. High-fat and high-fructose diets are frequently associated with oxidative stress; however, the development of detectable oxidative damage depends on the duration and intensity of the metabolic challenge, the tissue examined, and the sensitivity of the biomarkers used. One possible interpretation is that the 12-week experimental protocol captured an early stage in which structural and metabolic adaptations preceded a measurable disruption of renal redox homeostasis. Alternatively, oxidative changes may have occurred transiently, locally, or through pathways not detected by the biomarkers evaluated here. Therefore, the present results do not demonstrate that oxidative stress is absent from the pathogenesis of HFHF-associated renal injury; rather, they indicate that a generalized oxidative imbalance was not detectable using the evaluated endpoints at the time of tissue collection.

This observation is particularly relevant for interpreting the effects of jabuticaba addition in HFHF diet. Although *Myrciaria cauliflora* is widely recognized as a source of phenolic compounds, particularly anthocyanins concentrated in the peel, the beneficial effects observed in the present study cannot be attributed to antioxidant activity, as neither the HFHF challenge nor jabuticaba addition significantly altered the evaluated oxidative stress markers or antioxidant enzyme activities. Instead, the effects of jabuticaba were primarily associated with the preservation of renal histological architecture and modulation of the relative elemental profile. Beyond their antioxidant properties, jabuticaba and jabuticaba-derived products have demonstrated anti-inflammatory effects across different experimental models. As summarized by Inada et al. (2021), jabuticaba preparations have been shown to attenuate diet-induced intestinal inflammation by modulating inflammatory markers and reducing endotoxemia, alleviate experimentally induced colitis while preserving colonic architecture, decrease inflammatory mediators such as TNF-α in metabolic models, and reduce inflammatory markers in the hippocampus of animals exposed to a high-fat diet. Collectively, these findings support the anti-inflammatory potential of jabuticaba-derived bioactive compounds. Importantly, evidence of this potential has also been reported in the context of renal disease. Lima et al. (2025) showed that jabuticaba peel supplementation attenuated pro-inflammatory markers in patients with chronic kidney disease undergoing hemodialysis, further supporting the potential relevance of jabuticaba-derived bioactive compounds to renal protection.

The renal response varied according to the form of jabuticaba addition, highlighting the potential influence of food matrix composition and technological processing. The microencapsulated peel extract showed the most consistent histological response, with vascular congestion, inflammatory infiltration, and tubular vacuolization reaching values comparable to those of the Control group. This effect may be related to the fact that encapsulation has been shown to protect anthocyanins and other phenolic compounds from degradation, including under simulated gastrointestinal conditions, while enabling controlled release and potentially improving their bioaccessibility and bioavailability (Grgić et al. 2020). However, the relative contribution of improved compound stability versus enhanced systemic bioavailability cannot be distinguished from the present data, as circulating or renal tissue concentrations of anthocyanins and their metabolites were not measured. Therefore, enhanced bioavailability remains a plausible explanation for the more consistent histological response observed with HFME rather than a demonstrated mechanism.

The comparison between whole fruit and isolated peel further indicates that phenolic concentration alone may not determine the biological response. Whole jabuticaba provides phenolic compounds together with dietary fiber, minerals, carbohydrates, and other components of the natural food matrix, whereas the peel represents a more concentrated source of several phytochemicals. Dietary fiber and other components of the whole-fruit matrix may influence the gastrointestinal fate of phenolic compounds by affecting their bioaccessibility and interactions with the food matrix, while microbial fermentation may promote the release and metabolism of fiber-associated polyphenols, potentially modifying the generation and availability of bioactive phenolic metabolites (Bertelli et al. 2021; Rodríguez-Daza et al. 2021).

The analysis of the renal elemental profile provided additional evidence that metabolic overload affects renal tissue during the early stages of metabolic dysfunction. The kidney plays a central role in electrolyte homeostasis, and alterations in renal Na and Ca handling may reflect changes in tubular reabsorption and electrolyte transporter activity, processes that are closely linked to blood pressure regulation (Van der Wijst et al. 2018). In the present study, HFHF feeding altered the relative proportions of calcium, sodium, and manganese in renal cortical tissue, whereas potassium, magnesium, zinc, copper, and iron remained unchanged. Because SEM-EDS provides relative elemental composition rather than absolute tissue mineral concentrations, these findings should be interpreted as semi-quantitative differences in the elemental profile rather than direct measurements of tissue mineral concentrations.

The increase in the relative Ca proportion observed in HFHF animals may reflect altered elemental handling associated with metabolic imbalance. Calcium plays important roles in renal tubular transport, intracellular signaling, and renal vascular regulation (Staruschenko et al. 2024). Therefore, the differences in relative renal Ca composition observed among groups may reflect changes in renal mineral homeostasis, although they cannot be directly interpreted as alterations in Ca^2^⁺ signaling or transport. Importantly, however, the effects of jabuticaba addition in HFHF diet on Ca and Na were formulation-dependent and did not represent uniform normalization across treatments. HFWF shifted the relative proportions of Ca and Na toward the Control pattern. HFJP more clearly maintained the HFHF-associated pattern for these elements, whereas HFME showed an element-specific intermediate response. These differences may reflect the influence of food matrix composition and bioactive-compound delivery.

The alteration in Mn also deserves specific consideration. Mn was not selected a priori as an isolated biomarker of renal injury; rather, it was one of the elements detected as part of the broader SEM-EDS elemental analysis. Its biological relevance arises partly from its essential role in the catalytic activity of mitochondrial manganese superoxide dismutase (MnSOD/SOD2), a key enzyme involved in mitochondrial redox homeostasis (Grujicic and Allen 2025). Altered Mn levels have been associated with impaired renal function, including reduced glomerular filtration rate (Sánchez-González et al. 2015). Importantly, the increased relative Mn proportion observed in HFHF animals should not be interpreted as evidence of enhanced antioxidant activity, particularly because SOD activity and the other evaluated redox biomarkers remained unchanged. Instead, it may reflect altered elemental handling or tissue distribution during metabolic stress.

Taken together, the responses to jabuticaba were formulation-dependent, with the microencapsulated peel extract showing the most consistent improvement in renal histology, whereas whole-fruit and peel addition in HFHF diet produced distinct responses related to tissue organization and the renal elemental profile. These differential effects may reflect differences in food matrix composition and bioactive-compound delivery. The mild inflammatory infiltration and vascular alterations observed in the renal cortex further suggest that inflammatory and endothelial pathways may participate in early renal remodeling. Potential mechanisms underlying the effects of jabuticaba may therefore include modulation of inflammatory and endothelial pathways, as well as mechanisms involved in renal elemental homeostasis. These mechanisms would provide biologically plausible alternatives to a predominantly antioxidant effect. However, they remain hypothetical because inflammatory mediators, endothelial markers, renal transport proteins, and related signaling pathways were not directly assessed. Future molecular studies will be required to determine whether jabuticaba acts directly on these renal pathways or indirectly through systemic metabolic modulation.

The histochemical findings further support the interpretation that the model represents early rather than advanced renal remodeling. Increased PAS-positive material in the mesangial region of HFHF animals was consistent with mesangial matrix expansion, whereas Picrosirius Red staining did not reveal increased collagen deposition. Extracellular matrix remodeling is not restricted to fibrillar collagen deposition, as the renal ECM comprises multiple non-fibrillar components, including glycoproteins and basement membrane-associated proteins and proteoglycans. Notably, fibronectin accumulation has been associated with renal ECM remodeling, and fibronectin polymerization may precede collagen fibril assembly, supporting the concept that changes in ECM composition can occur without established fibrillar collagen accumulation (Biggs and Bradshaw 2025). The absence of increased Picrosirius Red staining therefore suggests that the structural alterations observed after 12 weeks had not progressed to established collagen-rich renal fibrosis. Interestingly, previous evidence in a diabetic mouse model showed that *Myrciaria cauliflora* extract attenuated glomerular injury, mesangial matrix expansion, and collagen deposition, supporting the potential of jabuticaba-derived compounds to modulate extracellular matrix remodeling in metabolically induced renal injury (Wu et al. 2016). Longer exposure to the HFHF diet could potentially result in more advanced extracellular matrix remodeling, but this remains to be tested experimentally.

The absence of collagen accumulation and major glomerular morphometric changes, together with the modulation of several structural alterations after jabuticaba addition in HFHF diet initiated following the induction phase, suggests that renal remodeling remained at an early and potentially modifiable stage. However, the present findings do not establish a specific window of reversibility. With prolonged metabolic exposure, progression toward tubulointerstitial fibrosis and loss of nephron function may reduce the capacity for structural recovery. Thus, the transition to established fibrosis may represent an important stage at which renal alterations become less amenable to nutritional modulation, although this hypothesis requires experimental confirmation.

From a translational perspective, the findings are relevant because metabolic disorders develop progressively, and renal structural alterations may emerge before clinically evident chronic kidney disease. Jabuticaba represents an interesting candidate for nutritional investigation because of its phytochemical content and its potential use as whole fruit, peel-derived ingredients, or technologically modified formulations. However, translation of the experimental jabuticaba dose to humans requires caution. The conversion of this value into a realistic amount of fresh jabuticaba would additionally require information on the polyphenol content of the specific fruit preparation and its bioavailability.

Although the present study provides relevant insights into the renal effects of early metabolic dysfunction and the potential protective role of *Myrciaria cauliflora*, several limitations should be considered. The experimental model represents an early stage of metabolic disturbance without established renal dysfunction, which may contribute to the absence of pronounced oxidative alterations and limits extrapolation to more advanced stages of kidney injury. Renal function was not directly assessed using serum creatinine, urea, cystatin C, albuminuria, or proteinuria; therefore, the observed histological preservation should not be interpreted as direct evidence of improved renal function. Inflammatory cytokines, endothelial markers, and molecular pathways potentially involved in the observed responses, including NF-κB signaling and renal ion transporters, were also not evaluated. Additionally, SEM-EDS provides relative elemental composition rather than absolute mineral concentrations, limiting quantitative interpretation of the elemental findings. Future studies using longer or more severe metabolic challenges and integrating renal functional biomarkers, inflammatory and endothelial mediators, molecular signaling pathways, transporter expression, and quantitative elemental analyses will be important to clarify the mechanisms underlying these early renal responses and determine whether jabuticaba addition in HFHF diet can prevent progression toward established kidney injury.

In conclusion, a high-fat, high-fructose diet induced early renal alterations characterized predominantly by vascular and tubular changes, inflammatory infiltration, and alterations in the renal elemental profile, without detectable oxidative imbalance or advanced collagen deposition. *Myrciaria cauliflora* addition in HFHF diet was associated with preservation of renal histological architecture and modulation of the elemental profile in a formulation-dependent manner, with the microencapsulated peel extract showing the most consistent histological effects. Overall, the observed protective response was primarily histological and associated with modulation of the renal elemental profile rather than antioxidant activity. However, confirmation of nephroprotective effects and identification of the underlying mechanisms will require further studies incorporating functional renal biomarkers and molecular analyses.

## Data Availability

No datasets were generated or analysed during the current study.
